# Supplementary material for: Diet-responsive transcriptional regulation of insulin in a single neuron controls systemic metabolism
Source: PLoS Biol. 2022 May 20;20(5):e3001655. doi: 10.1371/journal.pbio.3001655 (PMC9162364; doi:10.1371/journal.pbio.3001655)
Supplement: S1 Table — Strain names and genotypes of the C. elegans strains used in this study. (DOCX) [file pbio.3001655.s007.docx]

S1 Table. Strains used in this study

Strain names and genotypes of the *C. elegans* strains used in this study.

| **Strain name** | **Genotype** |
| --- | --- |
| Bristol strain N2 | wild-type |
| RJP4295 | *rpEx1752[ins-1p::NLS-GFP,rol-6]* |
| RJP5206 | *che-3(e1379); rpEx1752[ins-1p::NLS-GFP,rol-6]* |
| RJP4711 | *unc-13(e51)*; *rpEx1752[ins-1p::NLS-GFP]* |
| RJP4710 | *unc-31(e169)*; *rpEx1752[ins-1p::NLS-GFP]* |
| RJP5106 | *unc-13(s69); rpEx1752[ins-1p::NLS-GFP,rol-6]* |
| RJP5105 | *unc-31(e298); rpEx1752[ins-1p::NLS-GFP,rol-6]* |
| RJP4961 | *rpIs163(gcy-33p::unc-31 RNAi)*; *rpEx1752[ins-1p::NLS-GFP]* |
| RJP3826 | *rpEx1660[ins-1p::NLS::GFP,rol-6]* line 1 |
| RJP3934 | *rpEx1684[ins-1p::NLS::GFP,rol-6]* line 2 |
| RJP4411 | *ets-5(tm1734)*; *rpEx1660[ins-1p::NLS::GFP,rol-6]* |
| RJP3936 | *rpEx1686[ins-1pΔETS1::NLS::GFP, rol-6]* line 1 |
| RJP3937 | *rpEx1687[ins-1pΔETS1::NLS::GFP, rol-6]* line 2 |
| RJP3939 | *rpEx1689[ins-1pΔETS2::NLS::GFP, rol-6]* line 1 |
| RJP3940 | *rpEx1690[ins-1pΔETS2::NLS::GFP, rol-6]* line 2 |
| RJP3942 | *rpEx1692[ins-1pΔETS1ΔETS2::NLS::GFP, rol-6]* line 1 |
| RJP3943 | *rpEx1693[ins-1pΔETS1ΔETS2::NLS::GFP, rol-6]* line 2 |
| RJP3988 | *ets-5(nu1646)* – ETS-5::GFP CRISPR line |
| RJP567 | ynIs64(*flp-17::GFP*) |
| MH1317 | kuIs29(*egl-13::GFP*) |
| RJP4124 | *rpIs142(gcy-33p::mCherry, elt-2::GFP)* |
| RJP3127 | *ynIs37[flp-13::GFP]* |
| RJP255 | *ynIs34[Promflp-19::gfp]* |
| RJP3717 | *ins-1(nj32)IV* |
| RJP3782 | *ins-1(tm1888)IV* |
| RJP235 | *ets-5(tm1734)X* |
| RJP3774 | *ins-1(nj32)IV; ets-5(tm1734)X* |
| CX11697 | *kyIs536; kyIs538* (BAG ablation strain) |
| RJP4778 | *ins-1(nj32)IV; kyIs536; kyIs538* |
| RJP3886 | *ins-1(nj32)IV; rpEx1661[ins-1p::ins-1::SL2::GFP, myo-2p::mCherry]* |
| RJP3949 | *ins-1(nj32)IV; rpEx1696[ins-1pΔΔETS::ins-1::SL2::GFP, myo-2p::mCherry]* |
| RJP4013 | *ins-1(nj32)IV; rpEx1723[flp-17p::ins-1::SL2::GFP, myo-2p::mCherry]* |
| RJP3979 | *ins-1(nj32)IV, rpEx2101[ttx-3p::ins-1,myo-2p::mCherry]* |
| RJP4372 | *rpEx1723[flp-17p::ins-1::SL2::GFP* |
| RJP4207 | *daf-2(e1370)* |
| RJP3996 | *daf-2(e1370); ins-1(nj32)* |
| RJP4422 | *daf-2(e1370); ins-1(nj32);Ex1723[flp-17p::ins-1cDNA]* |
| RJP4208 | *daf-2(1370*); *rpEx1790*[*ges-1p::daf-2a, myo-2p::mCherry*] |
| JN1723 | *daf-2(e1370)*; *peEX1723*[*H20p::daf-2a, myo-3p::venus*] |
| RJP4318 | *daf-2(e1370); ins-1(nj32)*; *peEx1723[H20p::daf-2a, myo-3p::venus]* |
| RJP4877 | *daf-2(e1370); ins-1(nj32)*; *rpEx1790[ges-1p::daf-2a, myo-2p::mCherry]* |
| RJP5325 | *daf-16(mu86); rpEx1723[flp-17p::ins-1::SL2::GFP, myo-2p::mCherry]* |
| RJP5265 | *ot853[daf-16::mNG::AID]* |
| RJP5266 | *ot853[daf-16::mNG::AID];ins-1(nj32)* |
| RJP5205 | *ins-1(nj32); muIS84[sod-3::GFP]* |
| *CF1553* | *muIs84[sod-3::GFP]* |
