# Supplementary material for: Diet-responsive transcriptional regulation of insulin in a single neuron controls systemic metabolism
Source: PLoS Biol. 2022 May 20;20(5):e3001655. doi: 10.1371/journal.pbio.3001655 (PMC9162364; doi:10.1371/journal.pbio.3001655)
Supplement: S1 Text — (DOCX) [file pbio.3001655.s010.docx]

Supplementary Methods

Transgenic lines

All constructs were injected into young adult hermaphrodites as complex arrays. *ins-1* rescue constructs were injected into *ins-1(nj32)* background: 2 ng/µl of construct (*ins-1p::ins-1::SL2::GFP*, *ins-1pΔETS1ΔETS2::ins-1::SL2::GFP, flp-17p::ins-1::SL2::GFP*) were injected with 5 ng/µl of *myo-2p::mCherry* and 180 ng/µl bacterial DNA. While the GFP of the rescue construct was visible in some cases, the rescuing concentrations used were too low to reliably see GFP expression. *ins-1* promoter reporter assays: 50 ng/µl of construct (*ins-1p::NLS::GFP*, *ins-1pΔETS1::NLS::GFP, ins-1pΔETS2::NLS::GFP, ins-1pΔΔETS::NLS::GFP)* injected with 50 ng/µl *rol-6* co-injection marker and 180 ng/µl bacterial DNA. Microinjections were performed using standard methods(*1*).

CRISPR/Cas9

The endogenously tagged ETS-5::GFP strain was generated by Knudra Transgenics (now NemaMetrix). Genomic modifications include: The last 34 amino acids of ETS-5 are recoded with silent mutations to prevent recutting. GGGGSYG linker with intron inserted, and codon-optimised eGFP inserted C-terminally. TGTGSGSSTS linker with intron containing the loxp flanked unc-119 rescue cassette and stop codon.

Molecular cloning

***ins-1p reporter construct:*** A genomic fragment was PCR-amplified from the first codon of the *ins-1* gene to 2.574 kb upstream. The forward primer incorporated a HindIII site, and the reverse primer incorporated a BamHI site. The HindIII and BamHI digested promoter fragment ligated into HindIII and BamHI digested pD95.67 vector, resulting in *ins-1p::NLS::GFP****.* Promoterless *ins-1cDNA* construct*:*** To generate a construct containing *ins-1* cDNA that would enable insertion of multiple promoter fragments: *ins-1 cDNA* (330 bp) was amplified from N2-derived cDNA incorporating NheI and KpnI sites. The *ins-1 cDNA* insert was cloned using NheI-KpnI into MCS2 of the pSM_SL2_GFP vector. ***ins-1p::NLS-GFP ETS-site* mutagenesis*:*** The ETS sites within the *ins-1p::NLS-GFP* reporter construct were mutated using site-directed mutagenesis. First, individual sites were modified independently and sequenced, then the *ins-1pΔETS2::NLS-GFP* construct was used for site-directed mutagenesis of ETS1 to generate *ins-1pΔΔETS::ins-1::SL2::GFP.****ins-1p::ins-1cDNA* rescue construct*:*** The *ins-1* promoter was PCR amplified from the *ins-1p::NLS-GFP* construct, incorporating SmaI sites to non-directionally clone into MCS1 of the pSM_ins-1cDNA_SL2_GFP vector. Directionality was confirmed by sequencing. ***flp-17p::ins-1cDNA* rescue construct*:*** A 200 bp fragment of the *flp-17* promoter was cloned into the pSM_ins-1cDNA_SL2_GFP by restriction-free cloning(*2*). The flp-17 promoter was amplified by PCR from *Prom9flp-17::mCherry*(*3*). ***ins-1p::ins-1cDNA ETS-site* mutagenesis*:*** The *ins-1pΔΔETS::ins-1cDNA* construct was generated as described for *ins-1pΔΔETS::ins-1::SL2::GFP* above, sequentially mutating ETS2 then ETS1 using *ins-1p::ins-1cDNA* as a mutagenesis template.

Primers:ins1p_ETS1_SDM_F catttatgaaaaatcaatttttaaggacacggtctctttgaacac,

ins1p_ETS1_SDM_R gtgttcaaagagaccgtgtccttaaaaattgatttttcataaatg,

ins1p_ETS2_SDM_F cgaattcagggctatcgaactgtttaaggtaagcgc,

ins1p_ETS2_SDM_Rgcgcttaccttaaacagttcgatagccctgaattcg

Exploration assay

Cultures for exploration assays were maintained in low-population density, non-starved condition for at least two generations prior to commencing experiments. On day one of the assay, five L4 animals were placed on NGM plates freshly seeded with OP50 and cultured at 20ºC for four days. In parallel on day one, unseeded NGM plates were taken from 4ºC storage and placed at room temperature in stacks of two, to ensure even drying. On day three, a single colony of OP50 from a freshly streaked (within two weeks) plate was used to inoculate 400 mL of LB, which was incubated for 16 hours at 37ºC without shaking, then placed at 4ºC until required. In the afternoon of day four, each NGM plate was uniformly seeded with 400 µL of the OP50 culture, and left at room temperature until required for the assay. On day five at 9am 25 Christmas-tree L4 larvae from each genotype, were picked to fresh NGM+OP50 plates. At 5pm on day five, single animals were transferred to the centre of a uniformly-seeded plate, placed on the lab bench, and left undisturbed for 16 hours. Twenty worms were tested for each genotype, for each assay, and stacked 2 plates high during the assay. After 16 hours (day six of the experiment) worms were removed from the assay plates and the number of squares the tracks in the OP50 enter was counted (4.05 inches/line, 86 squares maximum). Exploration assays were repeated in biological triplicate on different days, always in parallel with a wild-type control, and blinded prior to counting squares entered.

RNAi experiments

RNAi of *pod-2* exploration assay was performed as **exploration assay** above with the following differences: On day one L4s animals were placed onto RNAi plates uniformly seeded with L4440 (empty-vector) bacteria. RNAi exploration plates were incubated at 37ºC for 16 hours, 24 hours before the exploration assay (day four). In parallel, 5 mL bacterial cultures of L4440 (empty-vector) or L4440_*pod-2* RNAi-containing HT115 *E. coli* were grown in LB + Ampicillin. After the 16 hours drying (day 5), the RNAi plates were uniformly seeded with L4440 or *pod-2* RNAi bacteria by coating the plates 2-3 mL of bacterial culture, tipping the culture off, then removing excess culture by flicking the plate sharply 3 times onto paper towel. The plates were then incubated at 37ºC for 7 hours, then moved to room temperature for one hour before beginning the exploration assay. Christmas-tree L4s were picked to the required RNAi plates (made previously) 8 hours before beginning the exploration assay, resulting in animals being exposed to the RNAi for a total of 24 hours for the assay. RNAi of *daf-16* followed by ORO staining was performed by placing synchronised L1s on *daf-16* RNAi plates or L4440 plates are prepared above. Animals were grown for 40 hours at 20ºC then transferred to 25ºC 24 hours prior to staining. For the glucose feeding experiments, animals were washed from the plates at 20 hours, then re-plated to either standard OP50 +H2O plates or OP50+40mM glucose plates 24 hours prior to staining.

Oil Red O staining detailed protocol

**Worm synchronisation:** Five L4s were picked to five fresh NGM+OP50 plates per genotype, and allowed to grow for 5 days. Worms were washed from the plates in 4 mL of M9 buffer into a 15 mL conical tube (Thermo Scientific) and eggs were isolated by adding 0.5 mL bleach (White King Premium) + 0.5 mL 5 M NaOH, mixing and incubating for 4 minutes. M9 was added to fill the tube, then samples were centrifuged for 1 minute at 1000 RCF, and the supernatant removed. Egg pellets were washed a further 3 times in M9 buffer, centrifuging for 1 minute at 1000 RCF. The egg pellet was resuspended in ~1 mL M9 and passed through a 40 µm filter to a fresh 15 mL tube. Samples were kept at room temperature, with gentle rocking for 24 hours to allow all worms to hatch and reach the L1 stage. Approximately 600 L1s were plated onto fresh NGM+OP50 plates (~8 plates per genotype), and incubated at 20ºC for 64 hours to reach young adulthood. **Staining:** Oil Red O stock solution was prepared by adding 0.5 g Oil Red O with 100 mL isopropanol. Solution covered with foil and mixed at room temperature for 2 days. Immediately prior to commencing fat staining, Oil Red O stock solution was diluted to 60% with sterile milli-Q filtered water, covered with foil and rotated at room temperature until required. Worms were washed from plates with 10 mL PBS and placed into a 15 mL conical tube using a glass Pasteur pipette, and centrifuged at 3300 RCF for 30 seconds. Supernatant was aspirated to 1 mL volume, worms were resuspended and transferred, with Pasteur pipette, to a 1.5 mL microcentrifuge tube. Worms were centrifuged at 3300 RCF for 30 seconds, supernatant removed to 100 µl volume, and pellet resuspended in 1mL PBS with a brief, gentle vortex to mix. Centrifugation and PBS wash steps were repeated a total of 2 times, finishing with an additional of 1 mL PBS and centrifugation. The samples were incubated on ice for 10 minutes, then supernatant was aspirated to 0.1 mL, 50 µL PBS, and 150 µL fixation buffer (30 mM PIPES, 14 mM Na_4_EGTA, 40 mM NaCl, 160 mM KCl, 1 mM Spermidine, 0.4 mM Spermine, 4 % Formaldehyde, 0.5 % β-mercaptoethanol). Samples were fixed for 5 minutes at room temperature, mixing gently during fixation. Placing tubes in a floating rack, the tubes were placed in a dry ice + ethanol bath for 3 minutes, ensuring contents of tubes were completely frozen. Samples were then partially thawed by placing at room temperature water for 1 minute. Samples were immersed in the dry ice + ethanol bath for 2 minutes, thawed in room temperature water for 1 minute, and again immersed in the dry ice + ethanol bath for 2 minutes. Samples were thawed at room temperature for 45 seconds, then tubes were quickly, and with moderate pressure, swiped across a tube rack to obtain an ice slurry. Tubes were laid down at room temperature until the ice slurry completely melted (5-10 minutes). Samples were centrifuged 3300 RCF for 30 seconds, supernatant aspirated to 100 µl, 1 mL PBS added then briefly vortexed. Wash steps were repeated for a total of three washes. After removing the final supernatant to 100 µl volume, 1mL of 60 % isopropanol was added to the worm pellet, and samples were incubated for 10 minutes with rotation at room temperature. Samples were centrifuged 3300 RCF for 30 seconds and maximum supernatant was removed without disturbing the worm pellet. Oil Red O working solution was filtered through a 0.22 µm filter, then 400 µl was added to each worm pellet. Samples were covered in foil, then incubated with rotation, overnight at room temperature. The following day, the stained worms were washed twice in PBS + 0.01% Triton-X100, then washed once in PBS, centrifuging 3300 RCF 30 seconds. Supernatant was removed, then worms were resuspended and mounted onto agarose pads for imaging.

Chromatin Immunoprecipitation – qPCR

**Sample preparation:** ChIP-qPCR of ETS-5-GFP was performed on non-starved synchronised L4 animals collected as follows. On day one, approximately 100 L4 hermaphrodites were picked to fresh NGM+OP50 plates. Day two, 15 animals (now young adult) were transferred to fresh NGM+OP50 plates and allowed to lay eggs for four hours, then removed. Eggs were incubated at 20ºC for 67 hours (~10 eggs/adult stage). Animals were washed from NGM plates with M9 buffer, and incubated with 2.5 mL bleach and 2.5 mL 5 M NaOH for four minutes to release eggs. Eggs were then washed 3-4 times in M9 buffer, centrifuging at 1000 RCF, 1 minute between wash steps. Eggs were plated at a density of ~600/plate to fresh NGM+OP50 plates. 62-63 hours after egg plating, late L4s were washed off plates with M9, centrifuged at 1000 RCF for 1 minute, and washed two times in M9. L4s were then washed once in PBS + protease-inhibitor complex (PIC). L4s were then resuspended in 1mL PBS + PIC and snap frozen as droplets in liquid nitrogen (“popcorn”) that were stored at -80ºC until required. **Chromatin preparation:** To prepare chromatin: frozen popcorn was ground with a mortar and pestle on dry ice to a fine powder. The frozen powder was transferred to a glass Dounce homogenizer containing 20 mL PBS + PIC + 1.1% formaldehyde. Samples were cross-linked at room temperature for 10 minutes - during this time the samples were ground using the tight “B” pestle. 1 mL of 2.5 M glycine was added to the sample to quench the formaldehyde for 5 minutes at room temperature, while still homogenizing the sample. Samples were then centrifuged for 5 minutes at 6000 RCF, the supernatant removed and pellet washed once with PBS+PIC. The centrifugation step was repeated, supernatant removed and the pellet resuspended in 500 µl FA buffer (50 mM HEPES, pH 7.5, 1 mM EDTA, 1% Triton-X100, 0.1% Sodium deoxycholate, 150 mM NaCl, PIC). Samples were sonicated using a Covaris S220 sonicator in 150 µl tubes using the following settings: Duty cycle 2 %, Intensity 6, cycles/burst 200, for 12 minutes. After sonication of all samples was complete, they were centrifuged at full-speed for one minute (room temperature), the supernatant was kept on ice and the pellet resuspended in 150 µl FA buffer and sonicated an additional time under the same conditions. All sonicated samples were pooled, centrifuged at maximum speed at 4ºC for 10 minutes. The supernatant was collected, DNA concentration measured using a nanodrop, and frozen at -80ºC until required for Chromatin immunoprecipitation (ChIP). **Chromatin Immunoprecipitation**: 20 µg of chromatin was made to 650 µl volume with FA buffer in a low bind microcentrifuge tube (Eppendorf). Samples were incubated with 2 µl of α-GFP antibody (ab290 abcam) with overnight rotation at 4ºC. For each sample, 10 µl Protein-G dynabeads (Invitrogen) was added to a fresh low bind tube, and washed once in 1 mL FA buffer. 500 µl chromatin/antibody sample was added to the beads, and 50 µl chromatin/antibody sample was kept in a fresh low bind tube as input (10%). Samples were incubated with beads for 3 hours, with rotation at 4 ºC. Beads were then washed 2 times with 1 mL FA buffer, with 5-minute rotation at 4 ºC. Then washed first with 1 mL FA-1M buffer (FA buffer with 1 M NaCl), then LiCl buffer (250 mM LiCl, 1% NP-40, 1% sodium deoxycholate, 1 mM EDTA, 10 mM Tris-HCl, pH 8) for 10 minutes with rotation at 4ºC. Finally, beads were washed twice, quickly with TE Buffer then resuspended in 100 µl TE. 50 µl TE was added to input samples. Tubes were then wrapped in parafilm, and incubated overnight at 65ºC with 1000 rpm shaking. **DNA purification**: 1 µl RNAseA (Qiagen) was added to each sample, then incubated at 37ºC for 30 minutes with shaking. Then, 5 µl 10% SDS and 1 µl 10 mg/mL Proteinase-K solution was added to each sample, and incubated 55ºC for 1.5 hours with 1000 rpm shaking. DNA was then purified using the Qiagen min-elute kit as follows: 600 µl PB was added to each sample, mixed thoroughly by pipetting. Tubes were then placed on a magnetic rack to clear beads from solution. All sample was then added to mini-elute columns, centrifuged 1300 rpm 1 min. Columns were washed 2x with 600 µl PE buffer, then columns dried by a further 2-minute centrifugation. 20 µl EB buffer was added to the column, left to stand for 2 minutes, then centrifuged 1 minute to elute the DNA. **qPCR analysis**: ChIP and input samples were diluted 1:10 in PCR-grade water (Roche), and 4 µl of each diluted sample was pipetted in triplicate and mixed with 1 µl 10 µM primer mixture for control region or *ins-1* promoter ETS region, and 5 µl SYBR green (Roche) and analysed using the Light Cycler 480 (Roche). Primers: ins-1_ETS_ChIP_F: aggacacggtctctttgaaca, ins-1_ETS_ChIP_R: cctgaattcgccataaaacg, ins-1_Control_ChIP_F: gtaggcaaaaatgcctctgc, ins-1_Control_ChIP_R: gattaggggtggcgataggt

Western Blot

Protein gels were made using the mini-PROTEAN system (BioRad). Eight non-synchronised, non-starved plates of worms were washed off in M9. Centrifuged at 1700 rpm, washed 3 times in M9, and the supernatant removed. Samples were transferred to 1.5 mL micro-centrifuge tubes, then 50µl loading buffer was added and samples boiled for 5 minutes. Samples were centrifuged and full amount was loaded into the wells of a 10 % Tris-Cl acrylamide gel, including 10 µl protein standard ladder (Novex). Gels were run for 45 minutes at constant mAmps of 0.04. Proteins were transferred to PVDF membranes using iBlot ministacks (Invitrogen) and iBlot device (Life technologies), 20 V 7 minutes. Membranes were blocked in TBST (20 mM Tris, 120 mM NaCl, 0.05% Tween 20) + 5% skim milk powder, for 1 hour at room temperature. Membrane was then incubated with ab290 1:1000 diluted in TBST+5% skim milk powder for 1 hour. Membrane was then washed 3 x 10 minutes in TBST. The membrane was then incubated at room temperature with HRP-conjugated α-Rabbit IGG 1:5000 in TBST + 5% skim milk powder. Membrane wash steps were repeated at previously, then the membrane was incubated with ECL western blotting substrate (Pierce/Thermo Scientific) solution for 2 minutes and imaged using ChemiDoc MP imaging system (Biorad).

**References**

1. C. C. Mello, J. M. Kramer, D. Stinchcomb, V. Ambros, Efficient gene transfer in C.elegans: extrachromosomal maintenance and integration of transforming sequences. *Embo J* **10**, 3959-3970 (1991).

2. S. R. Bond, C. C. Naus, RF-Cloning.org: an online tool for the design of restriction-free cloning projects. *Nucleic Acids Res* **40**, W209-213 (2012).

3. J. P. Brandt *et al.*, A single gene target of an ETS-family transcription factor determines neuronal CO2-chemosensitivity. *PLoS One* **7**, e34014 (2012).

4. R. Lorenzo, M. Onizuka, M. Defrance, P. Laurent, Combining single-cell RNA-sequencing with a molecular atlas unveils new markers for Caenorhabditis elegans neuron classes. *Nucleic Acids Res* **48**, 7119-7134 (2020).
