# Supplementary material for: Diet-responsive transcriptional regulation of insulin in a single neuron controls systemic metabolism
Source: PLoS Biol. 2022 May 20;20(5):e3001655. doi: 10.1371/journal.pbio.3001655 (PMC9162364; doi:10.1371/journal.pbio.3001655)
Supplement: Attachment — Submitted filename: Response to Reviewers.docx [file pbio.3001655.s011.docx]

**Reviewer #1, Seung-Jae V. Lee: Handley et al., 2021, PLOS Biology**

In this manuscript entitled "Diet-responsive transcriptional regulation of insulin in a single neuron controls systemic metabolism", the authors showed that dietary glucose feeding from L4 stage significantly decreased ETS-5-mediated ins-1 expression in the BAG neurons and subsequently reduced intestinal daf-2 activity to increase DAF-16 expression. These series of reactions modulated foraging behavior and intestinal fat levels. This paper provides a new mechanistic insight into a discriminative role of insulin-like peptide in a single neuron and insulin/IGF-1 signaling in distal tissues under high dietary glucose conditions during post-developmental stage in C. elegans.

Major comment

1. The authors tested only one glucose concentration (40 mM) for glucose consumption. I wonder whether the downregulation of ins-1p::NLS::GFP by glucose is dose-dependent or not.

This is indeed an interesting question that examined prior to focussing with the 40mM glucose concentration. We performed preliminary experiments which suggested that lower glucose concentrations have the same effect on *ins-1* expression, shown here (n is not sufficient for correct statistical analysis).

These data suggest that the downregulation of *ins-1p::NLS::GFP* is not dose dependent. However, to make our findings more physiologically relevant, we chose 40mM as this concentration of glucose leads to internal glucose levels that are equivalent to a poorly controlled diabetes patient [Schlotterer et al. 2009], yet not so high that it causes excessive mitochondrial damage [Alcantar-Fernandez et al. 2018; Alcantar-Fernandez et al. 2019]. Therefore we do not think it is absolutely necessary to include data on the dose responsiveness of *ins-1p::NLS::GFP.*

2. In figures 4,5, and 6, for key experiments, the authors need to show the data with glucose. It will substantially increase the cohesiveness of the paper.

The high glucose diet (40mM) substantially increases intestinal fat levels, much higher than the increase observed for *ins-1* mutants. It is unclear what information would be gained by including glucose-fed fat staining data for each experiment. The key question is whether BAG *ins-1* signaling can alter how glucose is metabolised and stored as fat in the intestinal cells, which we have addressed with the experiment shown in Fig 7C. These data show that overexpressing INS-1 specifically from the BAG neurons is sufficient to significantly reduce intestinal fat storage on the high glucose diet.

3. For key fat staining experiments, they need to obtain the data with L-glucose as well as D-glucose. If the fat staining data are similar between these two, glucose sensing or glycation mediate this signaling. If the data are different between these two, nutrients and metabolism are important, suggesting the existence of additional metabolic signaling for fat regulation in addition to the signaling axis shown in this paper. I think both data will be interesting and will help this paper.

Fat accumulation on D-glucose and L-glucose are not expected to be the same as L-glucose is not metabolized. The fat accumulation on L-glucose would therefore be expected to be closer to the fat accumulation observed for the *ins-1* mutant, although both D- and L- glucose reduce INS-1 expression by ~50 % so the effect on L-glucose would not be expected to be as strong as the *ins-1* null mutant. We do expect D-glucose to have additional metabolic fat regulation in addition to the signalling axis shown in this paper. Consider the DAF-2/DAF-16 data presented in Figures 6 and 7 – where we show that the majority of fat accumulation on excess glucose is independent of DAF-16, from the text:

“*we found that DAF-16 is required for generating ~34 % of the fat accumulated over 24 hours on 40 mM glucose (Fig. 7C). This means that DAF-16 is not required for accumulating most (~66 %) of the fat increase observed when animals are fed 40 mM glucose for 24 hours. We therefore hypothesize that the BAG-INS-1 signaling pathway acts on a specific intestinal metabolic pathway that requires DAF-16, compared to the majority of glucose-induced metabolic changes that are DAF-16 independent.”*

We therefore do not feel that repeating the fat staining experiments with L-glucose is required.

Minor comment
1. In Fig. 4C, the quantification numbers are not sufficient to show the effect of ins-1 mutations on concentration of TAGs. They can perform additional sets or move the data to supplementary figures.

These data have now been moved to Supplementary Figure 5A.

2. In Fig. 6B, please add the data of the ins-1(nj32) single mutants at 25 °C.

The data for N2 and *ins-1(nj32)* at 25ºC are shown in Figure 6E, which demonstrates there is the same difference in fat accumulation between wild-type and *ins-1(nj32)* at both 20ºC and 25ºC. Fig 6B was performed with *daf-2(e1370)* as the control as this was the important comparison to make. As N2 and *ins-1(nj32)* animals grow at different rates to daf-2(e1370), N2 and *ins-1(nj32)* were not included as part of the experiment in 6B.

3. "As loss of ins-1 in the daf-2(e1370) mutant leads to an increase in fat storage at 20 °C, these data suggest that BAG-expressed INS-1 activates DAF-2, and when INS-1 is absent DAF-2 activity is decreased." is not consistent with the data in figure 4.

When DAF-2 activity decreases, animals store more fat. Figure 4 shows that loss of *ins-1* leads to animals storing more fat. In figure 6A DAF-2 (at 20ºC) is partially active – BAG ins-1 will be contributing to this activation. When INS-1 is removed, DAF-2 is even less active, therefore the animals store even more fat in the double mutant. The data are consistent with this hypothesis.

4. There is no speculation about why ins-1(nj32) mutants displayed stronger phenotype for lipid accumulation or exploration than ins-1(tm1888) mutants. Please discuss this issue by describing the allelic difference between ins-1(nj32) and ins-1(tm1888) mutants.

We have now added additional text to speculate why the *ins-1(nj32)* mutant displays a stronger exploration defect than the *tm1888* mutant, and also highlighted that the difference in fat accumulation is not significant between these alleles.

5. Minor typos, grammatical and spacing errors throughout the manuscript need to be corrected. Following are some examples.
Please add space between number and unit.
Please be consistent for "life span" and "lifespan".
Please be consistent for "antagonize" and "antagonise".

Spaces have now been added between number and unit, and the words “life span”, “antagonize”, “behaviour” and “signaling” are all now consistent spelling.

6. It seems sufficient to just write "mid-stage L4 animals" without using "Christmas-tree" for stage description.

We have now deleted “Christmas-tree” for the stage description.

7. In Fig. 2E, it will be better to arrange the bar graphs to be consistent with the order of results.

The bars are consistent with the order the results are presented. H_2_O and D-glucose are controls, therefore are placed as the first two bars. Then the remaining bars are presented in the order they are described in the text.

8. Please follow the C. elegans genetic nomenclature. For example, ins-1(nj32); daf-2(e1370) in Fig. 6 should be corrected to daf-2(e1370); ins-1(nj32).

These errors have now been corrected in Fig 4E (ins-1; ets-5) and Figs 6 and 7 (daf-2; ins-1).

**Reviewer #2:** In this manuscript, Handley et al uses C. elegans as a model system to address the role of insulin-like signaling in coordinating multiple physiological events such as fat storage and food-seeking behavior in response to excess dietary glucose. First, they found that excess dietary glucose decreases expression of ins-1 specifically in BAG sensory neurons and this regulation does not depend on neurotransmission or neuropeptide signaling and is maintained in che-3 mutants that are defective in cilia. Next, they showed that a transcription factor ETS-5 regulates ins-1 expression in response to excess glucose and the expression of ets-5 also decreases when dietary glucose level is high. They further showed that ets-5 binds to the promoter of ins-1 and thus causally linked these two molecular responses. Meanwhile they showed that ins-1 expression in BAG suppresses intestine fat storage and facilitates food-seeking movement. These results place ins-1 signaling in between external condition of high glucose and internal response of fat storage and food-seeking behavior. Next, they further showed that ins-1 signal from BAG antagonizes daf-2 receptor in the intestine to regulate fat storage and acts through daf-16 in the intestine in response to excess glucose. The results characterize the function of an insulin-like pathway in regulating fat metabolism and food seeking that contribute to our understanding of metabolic responses to glucose stress, a question that has important impacts on human health issues.

Overall, the experiments are well designed and carefully conducted. The results are compelling. I have a few suggestions that may help to improve the manuscript.
1.    The authors pointed out that they used a lower glucose concentration and a shorter consumption duration than those in several other studies and that these differences may be important for the characterization of the signaling mechanisms in this study. It seems that the glucose concentration is still high, and it will be informative if authors can further discuss various impacts that different levels of excess glucose concentrations may have on physiological processes and perhaps how cells differentially respond to these external stresses.

This is a very good point, the level of glucose is still quite high, because we wanted to use a level of glucose that has been shown to have some physiologically-relevant effect on the animals. In this case, that 40mM resulted in internal glucose levels that were comparable to a poorly controlled diabetes patient. While the concentration of glucose is still high, the treatment time is also much shorter than in other studies. We have now written additional discussion about this difference, as well as include our take on the very interesting idea of differential regulation in different cell types.

“*This glucose concentration is still high enough to induce life-span changes, mitochondrial damage, egg laying defects and lead to significant increases in fat storage. However, the …”*

*“The time frame of treatment used here is much shorter than previous studies and we expect that the time is short enough so that animals are able to mitigate the effects of excess glucose by activating the appropriate responses, yet is not so long that metabolic homeostasis has broken down. Our data show that animals still gain a significant amount of fat in the 24 hours on high glucose. The stress-response systems required to mitigate damage from this excess glucose are likely to be activated but not overwhelmed in this time-frame, as our data showing a small but significant SOD-3::GFP induction would suggest. One aspect for further study is to take into account that different cell types have different metabolic needs; therefore, we could expect that tissues other than the intestinal cells respond to excess glucose differently in this timeframe. Our readout of BAG-INS-1 function was changes to intestinal fat storage, yet it is possible that BAG-INS-1 also affects the metabolic programs of muscle and neurons – that there is differential metabolic reprogramming by BAG-INS-1 in other cell types is an intriguing possibility.”*

2.    Their experiment on POD-2 suggests that ins-1 signals to fat storage to regulate food seeking behavior. This is interesting and would be helpful if authors can discuss potential signaling pathways from intestinal fat storage to food-seeking movements.

This is a very interesting question, the potential signalling molecules from the intestine to neurons are many, but none have been specifically described for the glucose response. Insulin-like signalling is also likely to be involved in the intestine-neuron direction. For example, *ins-11* acts as a signal from the intestine to the sensory neurons to control avoidance behavior is response to pathogenic bacteria (Lee & Mylonakis, 2017). We also found that while intestinal DAF-2 expression rescued the intestinal fat deposition, this did not rescue the exploration behavior (not shown as this adds far too much complexity and is downstream of the fat deposition mechanism of BAG-ins-1). Suggesting that neuronal DAF-2 is required for correct feeding behavior – in agreement with previously published work (McCloskey RJ et al. 2017)

We have expanded the text to include the fat-regulatory molecules that were shown to be involved in modulating food-seeking behavior.

“…*with increased fat storage leading to decreased food-seeking behavior. This is regulated through the SREB-SCD pathway, an acetyl-coA carboxylase, and multiple nuclear hormone receptors*”

3.    It is possible that excess glucose affects the growth of OP50 which subsequently affects worm metabolism. Their experiments using L-glucose argues against this possibility. But, it will be very helpful if authors discuss this possibility and how much they think that the external glucose exerts the effect on worm metabolism directly or indirectly.

We thank the reviewer for this observation, this is a very good point. We have now added experimental data with heat-killed OP50 as Fig 2A, and added further discussion to address this comment.

*“The effect of glucose on OP50 growth, the BAGs or other neurons may sense glucose in the external environment, other neurons that sense metabolic changes may signal to the BAG neurons to regulate ins-1 expression, the BAG neurons may autoregulate ins-1 expression via autocrine signaling or the BAG neurons may directly sense internal increases in glucose. To test the effect of glucose on OP50 growth, we measured BAG ins-1p::NLS-GFP expression when OP50 was heat-killed prior to adding to the glucose plates. We found that BAG ins-1p::NLS-GFP expression was significantly decreased on the heat-killed bacterial plates (Fig. 2A), therefore alterations to OP50 growth or metabolites when grown on glucose do not contribute to BAG-ins-1 down-regulation.”*

4.    Mutants of cilia are often partially defective in sensing. It is not clear how much che-3 is defective in sensing and whether it is possible that ins-1 expression in BAG still partially depends on sensing external cues?

We have added an additional sentence to clarify this point: *“In this mutant the BAG, and other ciliated neurons have no connection to, and therefore cannot sense, the external environment.”*

5.    On page 16, they stated that "Here, we found that DAF-16 is required for generating ~27 % of the fat accumulated over 24 hours on 40 mM glucose (Fig. 7C). This means that DAF-16 is not required for accumulating most (>70%) of the fat increase observed when animals are fed 40 mM glucose for 24 hours." How did authors quantify the effect here? It may be helpful to explain in the methods if they have not.

Thank you for the comment, we have now added these calculations to the quantification and statistical analysis methods. Upon verifying these calculations, we found slightly different, and more specific estimated proportions of *daf-16* dependent and *daf-16* independent fat accumulation (34% and 66%, respectively). The text has now been updated.

6.    How does L-glucose have a similar effect as D-glucose? They very briefly discussed about this. This effect is not expected but interesting. It will be helpful if the authors can further speculate the mechanism.

This is a very interesting question, and one we did not want to speculate too much on as the possibilities are quite vast and not easily testable. To address this point, we have now added additional text in the discussion to further expand about how L-glucose has a similar effect as D-glucose:

*“Protein glycation plays an important role in the pathology of diabetes (Negre-Salvayre et al. 2009), and because it is non-enzymatic it can be achieved by both D- and L-glucose. Non-enzymatic glycation lacks sequence motif bias, so targets of this post-translational modification are difficult to predict, identify, and validate. The identity of the glycation target upstream of ins-1 regulation could possibly be ETS-5 or it may be another unknown molecule or molecules. Identifying upstream targets will be facilitated by the advancement of new technologies and methods for studying non-enzymatic glycation, and will be an important aspect of future work.”*

7.    In figS1D, it is very difficult to see what the authors are showing.

The micrographs have been enlarged and the intestinal cells have been outlined to make these images clearer.

**Reviewer #3:** Metabolic homeostasis is dependent on specific and conserved signaling pathways such as the insulin/insulin-like signaling pathway in metazoans. This work involves the use of C. elegans as a model to assess how exogenous glucose impacts signaling pathway in the sensory neurons. Authors convincingly determine:
*    Dietary glucose reduces INS-1 expression in the BAG glutamatergic sensory neurons.
*    INS-1 is controlled by the ETS-5 transcription factor which is also down regulated in response to dietary glucose.
*    INS-1 acts from the BAG neurons and not other INS-1 expressing neurons to inhibit fat storage via DAF-2.

Comments:
*    Authors show a schematic diagram of what ins-1 expression is expected to be in figure 5A- it is unclear if this pattern was experimentally determined.

We have now re-written this statement (below) to clarify how we determined the *ins-1* positive neuron identities. As BAG neurons were the only neurons that changed expression, we did not validate the identity of other neurons using reference reporter strains.

*“This ins-1p::NLS-GFP transcriptional reporter enables clear visualisation of INS-1-positive neurons, which we identified by neuron position, previously published expression patterns (Pierce et al. 2001) and single cell transcriptomic data (Cao et al. 2017; Lorenzo et al. 2020).”*

*    INS-1 expressed in BAG neurons is thought to activate DAF-2 and when INS-1 is absent DAF-2 activity is decreased. This is shown genetically but unclear if this is what is occurring in a glucose fed animal. If authors could make this more clear it would strengthen the manuscript.

We have now added additional text to integrate the endogenous DAF-16 expression data (Fig 7) with the genetic data: *“These data show that endogenous DAF-16 expression increases on the glucose diet corroborate our genetic observations that INS-1 modulates the DAF-2/DAF-16 pathway in response to glucose.”*

*    Most experiments assess the INS-1, ETS-5, Insulin signaling pathway. Authors don't quite integrate how a glucose diet directly impacts fat storage/exploration behavior and if such is indeed occurring through the ins-1; ETS-5 pathways within BAG neurons.

There are many other mechanisms that to respond to glucose, in addition to the neuronal mechanism we show here. Glucose induced fat-storage has been well established and involves many molecules – we have now included that glucose diet leads to fat deposition in the introduction.

… “have increased fat deposition (Lee et al. 2015; Mejia-Martinez et al. 2017) and…”

We have also added additional text to the discussion to reiterate that the BAG ETS-5/INS-1 pathway is a component of a more complex system.

*“Maintaining metabolic homeostasis is complex, and the response to excess dietary glucose is multifaceted including insulin-like signalling, TGF-β, SBP-1, and nuclear hormone receptors.”*

Increased fat stores affecting exploration/satiety behaviour has also been well established, including our previous work (Juozaityte et al. 2017) which demonstrated that feeding glucose induced satiety quiescence and reduced exploration behavior.

*    Transition between concepts were sometimes disjointed (eg from the idea that ins-1 mutant spent more time in a quiescent state and relation to ets-5 fat levels, pg 14). Introduce questions or hypothesis to provide rationale for next experiment.

We have now expanded the text to provide more rationale in this section.

*“Together, our data indicate that ins-1 mutants store more fat, which leads to increased satiety compared to wild-type animals grown on the same food source. Loss of ets-5 also leads to increased fat storage (Juozaityte et al. 2017), and we have shown that ETS-5 regulates ins-1 expression in the BAG neurons. To test whether ETS-5 and INS-1 act in the same pathway to regulate intestinal fat storage, we tested the intestinal fat levels of animals lacking both ins-1 and ets-5.”*

*    Minor comment- document would be easier to review if the page number and line number are placed on the document. If using a space between a number and the % sign make sure it is non-breakable; I think "30%" is more common than "30 %" but defer to the editor.

Spaces have been added between all numbers and units, as per Reviewer 1 comment, which is more conventional. Correct positioning will be examined upon reviewing the final proof.

*    Fig 7D needs a scale bar

Scale bars now added to this and experiments in supplementary Fig 6.

*    Can authors address if it is indeed the glucose and not a byproduct produced by the bacterial metabolism of glucose (perhaps use delta-PTS strain to double check the results shown in Fig 1B with OP50; as these results are foundational to the study.

We have now included new data in Fig.2A that addresses this question. We show that growing, then heat-killing OP50 prior to plating on glucose plates had the same result as live OP50, ruling out the possibility that a change in OP50 growth or metabolites is regulating *ins-1* expression.
